# Etymologia: Carbapenem

**DOI:** 10.3201/eid2507.ET2507

**Published:** 2019-07

**Authors:** Ronnie Henry

**Keywords:** carbapenem, antibiotics, antimicrobial dugs, broad-spectrum β-lactam antibiotics, thienamycin, imipenem, penicillins

## Carbapenem [kahr″bə-pen′əm]

A class of broad-spectrum β-lactam antibiotics, structurally similar to penicillins, with the substitution of a carbon atom (*carba*-) for a sulfur atom (Figure). This substitution creates a double bond on the pentane ring, which becomes a pentene ring (-*penem*).

**Figure:**
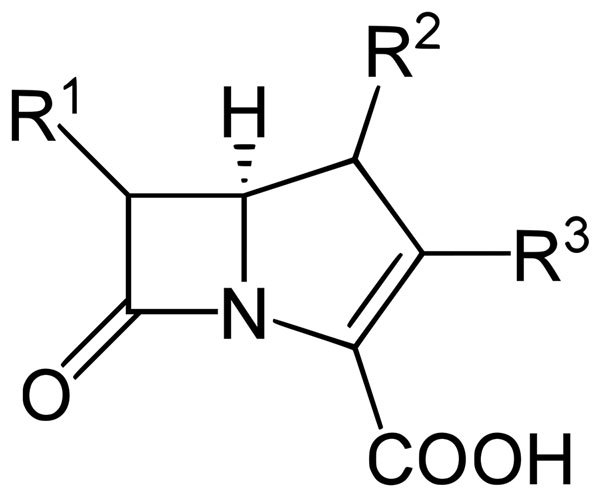
Backbone structure of a carbapenem.

The first carbapenem, thienamycin (*theion* [“sulfur”] + *enamine* [an unsaturated compound that forms the backbone of the molecule] + -*mycin* [suffix for drugs produced by *Streptomyces* spp.]), was discovered in 1976 in culture broths of the newly recognized species *Streptomyces cattleya*. Thienamycin rapidly decomposes in the presence of water, which limits its clinical utility.

The first carbapenem approved for use in the United States was imipenem, the stable N-formimidoyl derivative of thienamycin, in 1985. Resistance to imipenem, encoded on a mobile genetic element, was first identified in *Pseudomonas aeruginosa* in Japan in 1991, and carbapenemase-producing organisms have since spread globally.
